# Somaticization, the making and unmaking of minded persons and the fabrication of dementia

**DOI:** 10.1177/0306312719834069

**Published:** 2019-03-05

**Authors:** Alexandra Hillman, Joanna Latimer

**Affiliations:** WISERD, School of Social Sciences, Cardiff University, Cardiff, UK; Department of Sociology, University of York, York, UK

**Keywords:** dementia, diagnosis, somaticization

## Abstract

This article examines the strategies by which the different and variable signs of failing mental powers become known sufficiently for ‘dementia’ to be made into a stable bio-clinical entity, that can be tested, diagnosed and perhaps one day even treated. Drawing on data from ethnographic observations in memory clinics, together with interviews with associated scientists and clinicians, we document the challenges that clinicians face across the clinical and research domain in making dementia a stable object of their investigation. We illustrate how the pressure for early diagnoses of dementia creates tensions between the scientific representations of early dementia and its diagnosis in the clinic. Our aim is to highlight the extent to which the work of diagnosing dementia involves an intricate process of smoothing out seemingly insurmountable problems, such as the notoriously elusive connections between brain/mind and body/person. Furthermore, we show that a part of this process involves enrolling patients as minded, agentic subjects, the very subjects who are excluded from dementia science research in pursuit of biomarkers for the pre-clinical detection of dementia.

## Introduction

Characterized as an effect of degeneration, and as a temporal process of ‘unbecoming’ through which personhood is hollowed out and replaced by a recalcitrant ‘in-human being’, dementia is iconic of all that is most feared about growing old: loss of self, autonomy and personhood. While not the worst of human afflictions, its special character is one of becoming both ‘other’ and ‘unlovable’ (The President’s Council on Bioethics, 2005: 44). These matters are emphasized in social research. From Kitwood’s (1988) focus on *the person* with dementia to Beard’s (2016) call to recognize a life with dementia (rather than a living death), there are warnings about the multiple ways in which dementia creates conditions of possibility for someone to become perceived as a non-person (Behuniak, 2011).

Dementia is often portrayed as if it is an unproblematic and stable diagnostic category. As Lock (2011, 2014) points out, this is partly to do with the historical attribution of dementia as a ‘natural’ effect of growing older. In contrast, within the scientific and clinical communities themselves, dementia as a stable ‘bio-clinical entity’ (Keating and Cambrosio, 2003) is still emergent and its status ambivalent and uncertain (Moreira et al., 2009). Constituting dementia as a bio-clinical entity that can be investigated, known and treated involves epistemic shifts of dementia away from an association with old age.

Understanding this instability in dementia is particularly vital and made more complex in the context of the drive, in the UK as well as in other parts of Europe, for the early detection of dementia (e.g. DOH, 2016: 5). While dementia is a compendium category (George et al., 2011), there are attempts to refine its nosology through processes of differentiation. For example, the fifth edition of the Diagnostic and Statistical Manual of Mental Disorders (DSM-5) (re)inscribes dementia as a syndrome, a spectrum, and, a mental disorder. At the same time, it describes different types of dementia, each of which are locatable in different parts of the brain (American Psychiatric Association, 2013). This Includes increasing differentiation between different types of dementia: early and late onset alzheimer’s Disease (the latter being the most common form), Vascular dementia (the second most common form of dementia), Fronto-temporal Lobe dementia, Dementia with Lewy Bodies (which are tiny deposits of protein in nerve cells), and the dementias associated with Parkinson’s and Huntington’s diseases, and with Down’s Syndrome. Classificatory systems signify the increasing specificity inside as well as between each of these categories. For example, how Fronto-Temporal Lobe dementia is characterized by different kinds of behaviours, such as the loss of empathy, rather than cognitive function, helps make visible how each type reflects the sites in the brain affected. Importantly, summarizing dementia as a ‘neurocognitive disorder’, DSM-5 divides it into two categories – ‘major’ and ‘mild’ neurocognitive impairment, emphasizing that the threshold between the two is ‘inherently arbitrary’.

The advent and development of ‘mild (neuro)cognitive impairment’ (MCI) as a biomedical concept has helped mediate between laboratory neuroscience and the clinic, with the aim of creating one common understanding of dementia, and particularly of Alzheimer’s Disease (AD). Moreira et al.’s (2009) study of the production of MCI shows how the collective production of uncertainty amongst what they describe as the ‘bio-clinical collective’ was integral to the development of a purposefully contingent MCI category, an entity that could be transient enough to transport or bridge a variety of purposes and interests, including for example, policy drives for early detection.

Alongside differential diagnostic categories, a key way in which dementia is being stabilized, Lock (2014) argues, is through its increasing objectification and enactment as *somatic*. Finding ways to detect risk of dementia early – including identifying differences and mixed pathologies within specific disease categories – relies increasingly on their concreteness being located in organic and objective changes to different parts of the brain, rather than being established in their symptomatic expression. This includes relying on the creation of ‘biomarkers’ (Leibing, 2016) that are ‘organized around the visualization of pathology in the corporeal interior’ (Waldby, 2000: 24). As Leibing indicates, these biomarkers include those used in clinical research and those used in the clinic, or ‘diagnostic biomarkers’, which may or may not include genetic tests; there is overlap in biomarker use across research and clinical processes. Stabilizing dementia through its somaticization depends upon identifying biomarkers that offer ‘objective signs’ (Strimbu and Tavel, 2010) of dementia’s existence as a concrete object rather than a symptomatic entity.

The biomarkers increasingly being associated with dementia and that are most relied upon in research are located in brain tissue and are referred to as plaques (amyloid) and tangles (tau). These plaques and tangles can be seen in vivo on the new generation of brain scans. The amyloid and tau platform for dementia as a bio-clinical entity is being further stabilized through detection of genetic biomarkers such as Aβ_42_ and tau species in cerebro-spinal fluid (e.g. Sonnen et al., 2008). However, attempts to stabilize dementia diagnostic categories by detecting amyloid and tau biomarkers increases complexity in dementia research. This is because of the poor connection between symptoms characteristic of dementia (for example short-term memory loss, inability to learn new things, disorientation) and biomarkers of changes in brain pathology (Lock, 2014). Thus, dementia science is challenged to associate biomarkers such as plaques and tangles, or genetic biomarkers, with clinical symptoms (Leibing, 2016).

For some commentators, somaticization risks reducing human consciousness to ‘monism’, or ‘mind as body’ (Lock, 2014). As Moser (2011) helps illuminate, this creates a ‘somatizing’ mode of ordering that not only risks enacting the *dementia* as a matter of cognitive function, especially memory, locatable in the bio-physiology of the brain, but also enacts human subjectivity, the ‘I’, as a matter of cognitive function too – with personhood becoming a matter of brain and biology. A growing critical commentary on the expanding influence of ‘neuroculture’ in the context of ageing and dementia (Williams et al., 2011, 2012), emphasizes the importance of identifying when and how somaticization of mind is accomplished in dementia science, but also, when it is not (Pickersgill, 2013).

Translational medicine frameworks (the notion that medical innovation progresses in a linear way from bench to bedside) performs the ‘scientific’ domain as somehow discrete and of a different order from the ‘clinical’ domain. This has led many ethnographers to either look at the ‘research domain’ or the ‘clinical domain’. For example, Boenink (2016) looks across scientific discourses on AD biomarkers and research practices around a particular biomarker research project, while Laan (2016) does an ethnographic study of how biomarkers enter into a clinical practice setting.

The specificities of the current case, as is common in the UK, where ‘knowledge’ and ‘categorization’ are not yet secured enough to be sedimented, the clinicians in the clinics we observed are also researchers, or ‘clinician-scientists’ (Latimer, 2013). While we are not making any claim that the laboratory and the clinic are identical spaces, in tracking how ‘dementia’ is being fabricated (or not) we necessarily cross between scientific and clinical discourse and processes – this is an effect of the field as we have encountered it in our study of memory clinics. Because ‘content matters’ we focus on which biomarkers do or do not travel to the clinic with our clinician-scientists, and how they are or are not associated to stabilize (or not) a diagnosis of dementia.

We attend in what follows to the multiple ways in which dementia is brought into being both within *and* across the spaces of dementia science and research, clinical practice and the experiences of living with dementia. We show that there is a tension between the pursuit of biomarkers with which to detect dementia early and the clinical diagnosis of dementia, a tension which is at the heart of debates on and in dementia science as discussed above. Our examples from memory clinics suggest an interesting paradox. Attaching descriptions and experiences of cognitive decline, provided by patients and families, to diagnostic biomarkers may help to make dementia present (or not) as a bio-clinical entity. At the same time, these crossings between mind and brain depend upon clinicians invoking patients as minded persons, the very subject usually excluded from dementia science research in pursuit of biomarkers for the pre-clinical detection of dementia. Indeed we show how dementia is given form in the clinic through a process of assembling and associating heterogeneous materials with social processes, in ways that enact dementia as both somatic (located in a person’s brain) and relational. Somaticization is not a totalizing effect.

## The study

In this study, we draw upon our ethnography of dementia diagnosis carried out between 2012 and 2014. Fieldwork was undertaken in memory clinics based in two large Regional National Health Service hospitals attached to medical schools in the UK. Memory clinics have expanded in line with the global pressure from governments and public health policy for early detection technologies, the aim being to enable diagnosis of people *at risk* of developing dementia. However, the two memory clinics involved in the research were long-standing, established secondary services. They are both representative of hospital-based memory services across the UK and each functioned in similar ways, namely assessing patients experiencing problems with thinking and memory.

Like most services attached to large teaching hospitals, each memory clinic was set up by active clinician-researchers specializing in Alzheimer’s disease and other dementias. They were set up with two parallel purposes: to improve processes of assessment and diagnosis for those who may have dementia, and to support and develop research in the field in the context of pressure for early detection. Observations and interviews confirmed this dual purpose. Memory clinics are not only a site for early detection, they are an important resource for research and clinical trials recruitment.

In this dual capacity, memory clinics are places that sit at the boundaries of biomedical research and clinical practice, with many of their patients taking part in trials and other kinds of research and many of their clinicians working across these two settings. This study is therefore interested in the assessment, diagnosis and scientific study of dementia as a clinical context that extends the clinic into the laboratory and back again. As such, however, memory clinics also remain sites for the assessment and diagnosis of dementias – i.e. those people whose daily lives are already being affected by something that might be a form of dementia. The memory clinic is therefore also a site through which people are admitted, or not, to a diagnostic category such as AD.

The fieldwork involved the audio recording of and the taking of ethnographic fieldnotes at clinical consultations (N=51) and interviews with 13 memory clinic staff, 21 patients, 19 relatives/carers (ten of the patients and relatives were interviewed twice and one couple was interviewed three times). Additionally, ten biomedical scientists and researchers working in the dementia field were interviewed; these were all based in the UK, but were working within international collaborative networks in Europe and worldwide. Their areas of expertise included public health, genetics, base biology, clinical trials and a combination of these. They were identified through existing research and clinical practice networks, starting with names provided by the directors of the two memory clinic research sites, who were identified as having international influence in the field. Half of those in the sample were active in clinical dementia diagnosis and treatment, often working within memory clinics themselves, alongside their research practice. The researchers with whom we spoke represent a range of disciplines, which has a bearing on their perspectives regarding the science, detection and treatment of dementia; for example, our biologist’s interest in inflammation may not – of itself – be of central interest to the researcher in public health. However, the nature of their participation in research was similar. They tended to be involved in large, collaborative studies that brought different specialisms together. This was to a large extent because the overarching hypothesis of the dementia continuum, the necessity for early detection and the subsequent requirement for the identification of biomarkers or other kinds of risk indicators, was shared across different specialisms.

Our study shows clinics dedicated to diagnosis of dementia as being spaces made up of interactions between different kinds of clinicians (including nurses, doctors, psychologists and bio-science researchers), and between clinicians and patients and their families, through which dementia is (or is not) made concrete. A process of differential diagnosis is arrived at through the ‘assemblage’ (Latimer, 2013) of clinical tests, scans, cognitive tests, interpretation of patient histories and diagnosis; and it is in this process that other potential causes of memory or cognitive decline are ruled out (Bender, 2003).

Key themes were reflected in the assessment and diagnosis of those who were in the early stages or at risk of dementia as well as those with established dementia. We have chosen extracts that represent these key themes from the wider body of ethnographic material. We focus particularly on how dementia is stabilized within *and* across the spaces of dementia science and research, clinical practice and the experiences of living with dementia. We examine the practices through which scientists and researchers (who are often also practising clinicians) attempt to make dementia stable through its somaticization by binding concepts of dementia to epistemic objects such as biomarkers. We then explore the complexities that occur when these meanings cross over into clinics, where they collide with – as well as attach to – invocations of patients as minded subjects.

## Stabilizing ‘preclinical dementia’ and its imprecise association with biomarkers

Articulating the discursive alignments (Fujimura, 1987) of the sciences of dementia relies upon making dementia present as a biomedical entity through association with technologies and the objects that they produce as ‘biomarkers’. The proliferation of technologies include: instruments for brain scanning and methods of stimulation that permit the mapping and investigation of living brains (e.g. Shine et al., 2015), post-genomic technologies through which neurodegenerative diseases can be genetically profiled (e.g. Tanzi and Bertram, 2001), and experimental model systems that enable investigation and experimentation with living things, both human and non-human, for example Alzheimer’s mice (Giunta et al., 2008) and IPS cells (Milne, 2016).

The world forming around dementia science takes on its existence by way of how ideas, however imprecise, become bound to the ‘epistemic’ objects (Rheinberger, 2010) that technologies such as these help produce. Indeed, new biomedical classifications of dementia, including experimental research that attempts to establish aetiologies as well as genotypical and phenotypical profiles for different kinds of dementia, help to give dementia material presence. In short, dementia is granted its much-needed form, and its imprecision overcome, through binding dementia as a medical concept to the biomarkers amyloid and tau in humans (before any display of the symptoms of dementia).

The following extract taken from an interview with a dementia scientist illustrates this imprecision between biomedical concepts and epistemic objects.

Bioscientist 1: People in [his research] project *will have Alzheimer’s disease* – by definition – they’ll be biomarker positive, they’ll have evidence of amyloid and tau on their brain *but they don’t have dementia*. We’re trying to prevent dementia. (emphasis added)

The people taking part in research are defined as having Alzheimer’s disease but should not have any symptoms. In this scientist’s explanation, the *clinical* signs and symptoms of ‘the dementing patient’ are not just unnecessary but undesirable, because research is about prevention rather than cure. What gets cut out here is the human subject of dementia – the subject that displays the symptoms of dementia.

The need to work with people who have the biomarkers but not the symptoms is explained by another neuroscientist working in dementia:

Bioscientist 2: I mean it’s all based on the hypothesis that actually, and it may not be true, but it may be too late once the disease is well advanced. And our only hope really is around very early intervention. That’s where the smart money seems to be moving, towards very early intervention.

The need to establish biomarkers with which to detect dementia is for Bioscientist 2 where the ‘smart money’ is moving because, he says, early intervention is the only hope; it may be too late when the disease is well advanced. It is this hypothesis – that the greatest potential for success lies in intervening at the early stage when the triggers occur – that drives much of the research and health care policy in the field.

But are biomarkers enough to stabilize dementia? Bioscientist 1 suggests something more is needed than just biomarkers, if a diagnostic category of preclinical dementia is going to be firmed up, but adds that MCI (the condition in which some symptoms are already evident) is also not enough.

Bioscientist 1: MCI doesn’t exist, it’s a completely fabricated entity by a couple of guys in a room once over in the United States. It simply doesn’t exist. Because if you think about what makes a good diagnosis in medicine it’s **s***omething that tells you about aetiology, something that tells you about the response to treatment, something that tells you about the prognosis, something that tells you about the underlying path of physiology. MCI does none of those things. It’s completely nonsense.* (emphasis added)

At the same time as this researcher disparages MCI, he goes on to state how participants defined as having ‘pre-clinical’ Alzheimer’s disease are necessary to turn preclinical dementia into a bio-clinical entity. He goes on to say that to identify risk factors and potential disease modifiers, his research needs to be able to trace the neuro-pathological pathways of AD over time, by tracking the bodies of people identified as having plaques and tangles, amyloid and tau, however symptomless to begin with.

But MCI also provides a category of patient deemed at risk of dementia and who presents with traces of what might be dementia, as the following interview extract with a biological psychiatrist illustrates.

Interviewer: So you would see that perhaps [MCI] might contribute to – one of the major things that’s come up both in the clinic and in research from talking to people is knowing which of those cases you have of say mild cognitive impairment will go on to develop Alzheimer’s disease. And understanding more about that progression.

Biological Psychiatrist: Yes. So we’ve got a grant looking at exactly that. So we are looking at a cohort of 140 people with MCI and measuring markers of inflammation to see if that can explain why some go on to get Alzheimer’s early and others don’t. They are blood tests basically.

Here dementia is being firmed up through measuring markers of inflammation in patients with MCI and then tracking which patients go onto develop AD and so help bind AD to an epistemic object, the inflammatory marker as a biomarker of AD.

In these accounts of MCI we see first-hand the utility of MCI as what Moreira et al. (2009) identify as a ‘contingent category’: While MCI is fundamental to building the material presence of preclinical dementia for the continuation of dementia research, for biomedical scientists it also remains a flawed and weak concept, due to its poor prognostic capacity in relation to detecting dementia early enough. In other words, Bioscientist 1’s and the Biological Psychiatrist’s seemingly contradictory accounts present different versions of MCI and the opportunities that they offer. MCI’s contingency could equally position it as a ‘boundary object’ (Star and Griesemer, 1989), or grey zone (Droz Mendelzweig, 2009), that attempts to hold together the problematic relations between the world of neuroscience, the clinic and health policy pressure for early detection that presses somaticization of dementia.

In the research context, then, dementia is made matter, and so made to matter, by its increasingly becoming bound to epistemic objects such as ‘plaques and tangles’ or ‘inflammatory markers’. This binding of epistemic objects to different and imprecise concepts of dementia – ‘preclinical dementia’, ‘MCI’, ‘early’ – at the interface of neuroscience and medicine, includes patients with MCI most often identified and recruited through memory clinics. But studies that seek to observe biological mechanisms and test interventions or agents that could alter their course are designed on the basis that they can be and will be associated with symptoms of cognitive decline, something that has been increasingly problematized within the scientific community (Brayne, 2007; Humpel and Marksteiner, 2005).

As discussed, the approach to making dementia concrete through binding concepts (preclinical, MCI, early) to epistemic objects is seen to be somaticizing dementia, which is often seen as problematic. First, it makes for an increasing disjuncture between the science of AD and dementia (concerned with altering biological mechanisms), its clinical diagnosis (concerned with the functioning of the persons) and its everyday experience. Second, it risks making mind into something purely cognitive and locatable within the stuff of the brain.

As we show below, diagnosing dementia is as concerned with patients as subjects, and with the ongoing maintenance of personhood and its complex social and material relations, as it is with the attachment of these persons to different epistemic objects. Specifically, we show that it is precisely through the attachments made between forms of objectification (biomarkers) and attention to patients as persons that gives dementia form. This suggest that a somatic mode of ordering is only one of the strategies helping to stabilize dementia as a bio-clinical entity.

## Dementia in the clinic: Accounting for memory and the making of minds

The stabilization of dementia as a bio-clinical entity through processes of somatization runs into trouble because the subject of pre-clinical dementia – the patient and their bodily functions – should not display any signs or symptoms. At the same time, there is clinical evidence that people who are shown to have the biomarkers do not necessarily display any other signs or symptoms of dementia. In addition, while WHO (2015) loudly advocates that dementia is a ‘mental health issue’ and ‘not part of ageing’ (p. 1), biomedical research has correlated ageing and dementia for decades, making it a persistent ground of explanation amongst the medical community (Albert and Knoefel, 1994; Brayne and Calloway, 1988). Questions arise then from a clinical perspective over how and when patients who have somatic evidence of dementia also ‘bear witness’ to dementia as a bio-clinical entity.

In both memory clinics and following referral, usually by a patient’s family doctor, patients have an initial assessment. This assessment involves:

- cognitive tests, involving a combination of standardized questions and tests that are designed to test a person’s memory, their ability to learn new things, attention, reasoning, spatial awareness and language, commonly undertaken in a separate adjoining room by a psychologist or nurse practitioner but sometimes carried out by the doctor or psychiatrist as part of the consultation,
- the taking of a detailed patient history by asking questions of the patient themselves and their relative/carer, and
- clinical tests – some done on site that day, others arranged for a later date – including blood tests (mostly done to exclude any other potential clinical cause of their memory problem), a trace of the heart if it is a possibility that the patient may require medication for their memory which carries contraindications for some heart arrhythmias and, increasingly, a Computerized Tomography (CT) scan of the brain.

These assessments make available different forms of evidence, such as brain scans and cognitive test score results. Unlike in the research context, genetic biomarkers do not form a routine part of assessments for the diagnosis of dementia. There is a distinction made between the kinds of markers which are of clinical use and those that remain located in a research domain, as this clinician-researcher describes:

Bioscientist 3: Amyloid doesn’t particularly at all. So we know from fixed studies that you can have a lot of amyloid in the brain but you can be completely cognitively normal. So it’s just not specific enough … So people, that’s why I’m a little bit negative about any biomarkers because ultimately even on land, I mean if you look at it in isolation you can totally see why the clinician would say, that person looks to have dementia. They haven’t. They haven’t and that’s the bottom line.

While this same clinician-researcher described markers such as amyloid and tau as targets for research, forming part of the quest for identifying dementia risk, in the clinical context these markers are deemed to be of limited clinical utility. This is particularly the case when the clinical picture – particularly the presentation of the patient and their family – fails to provide the necessary symptomatic expression to connect these markers to a diagnosable dementia. That is not to suggest that attributions to the biological presence of dementia do not occur in the clinic; there is, for example, routine use of CT scans, in which alignments are made between cognitive test scores, patient accounts and the changes occurring in specific parts of the brain, as evidenced through the scan. In what follows, we examine how different objects and their interpretations are or are not brought into play in consultations with patients and family members when they are being diagnosed.

In our first extract from the memory clinics, we meet Mr and Mrs Smith. They are attending the clinic for the second time, following the results of a Computerized Tomography (CT) scan of the brain. Mrs Smith suffered a brain haemorrhage in midlife, from which she made a complete recovery. At the initial assessment, Mrs Smith completed cognitive tests. Following this assessment, she was sent for a CT scan. On return to the memory clinic, Mrs Smith is asked to do the same cognitive tests. The couple then meet with Dr Grey to discuss the test results.

Mrs Smith: So it’s just age, it’s my age?

Doctor Grey: Well no, you’re a bit more interesting than that. I think … [Dr Grey explains that there is scarring on the brain scan from Mrs Smith’s original stroke]. So the relevance is – in a way you have got less reserves than other brains would have, because for the last 20 years you’ve been doing very well on 90% function rather than 100% function. So if now we’ve got something extra, which there must be, because it’s a recent change, well, you’ve got less reserves than your husband would have or – in a way, I hate – I was going to say ‘normal’.

Mrs Smith: ‘Normal’, oh, I don’t mind.

Doctor Grey: A normal person of your age would have.

The focus of this interaction is on an object – the brain scan and the percentage of brain affected by changes. Doctor Grey situates the encounter in Mrs Smith’s previous history – that she has been functioning on less brain (90%) and therefore has less ‘reserves’.

This discursive trope of brain reserves reflects an important trajectory in neurosciences that is reconstituting the brain as much more ‘plastic’ than once thought (e.g. Belleville et al., 2011). But in Mrs Smith’s case it runs alongside the idea that her reserves are quantifiably less because of her earlier pathology – making her brain less able to ‘compensate’. He then goes on:

Doctor Grey: The two other things that the brain scan shows is that there is a bit of shrinkage of the brain over on this side which is well away from there. So just this area (he points at the scan), it’s opened, and there’s a bit of shrinkage in that part of the brain. Now that can happen with age … And then the one other thing is there’s this darker grey rim around it (points again). Now, that’s the sort of appearance you get when the smaller blood vessels are a little bit clogged up, which, again, is something that happens with age. So the bottom line is, yes clearly, the memory for new things – your husband was saying short-term memory, I think I’d probably say it’s more about learning new things being an issue, isn’t as strong as it should be … So probably it’s a combination of the fact that you’ve got less reserves because of that [the scarring] and so this smaller blood vessel problems and maybe that bit of shrinkage around there, is enough to be causing the problems. If this were firing on 100% than just on 90%, it probably would be much less of an issue.

The scan is a formidable ally that Dr Grey marshals as visual evidence, but it is the way that he juxtaposes the material evidence (the brain scan) alongside other evidence (the cognitive score, the history of stroke, the husband’s account) that firms up his interpretation: that the current changes to her brain (probably due to ageing) would be less remarkable in their effects if she had a ‘normal brain’ (and greater reserves) in the first place. This, it turns out, is why ‘she is more interesting’. The utilization of the brain scan, in this context, can be seen as a technology of ‘opening’, or a ‘document without end’ (Street, 2011), as it provides Dr Grey with the tools to describe multiple possibilities for the causes of Mrs Smith’s difficulties. Seminally, it also offers the potential for future re-interpretation of these possibilities, if and when Mrs Smith experiences any further changes to her symptoms.

As discussed, firming up the relationship between ‘normal’ aging and dementia has never been satisfactorily resolved. Yet, within the interactions taking place in memory clinics, ageing can be marshalled as an ally one moment and dismissed the next – as in the case of Mrs Smith.

Towards the end of this encounter Mr Smith expresses the ‘absent presence’ in the room:

Mr Smith: Let’s be honest. You (to his wife) were concerned about Alzheimer’s.

Doctor Grey: At the moment you wouldn’t fulfil any criteria for Alzheimer’s Disease whatsoever. But, first of all, I can’t foretell the future. The other thing is that Alzheimer’s Disease actually is quite common once you get into your seventies, eighties, but it progresses very, very slowly over eight, ten, twelve years. So I can’t tell you if we look 10 years ahead ….

The reassurance that this is not AD has required a complex set of negotiations to occur between the patient’s narrative, bringing the mind into presence through a subjective account of her cognitive failings, with interpretations of the cognitive tests and the CT scan. It is the associations between these various forms of evidence with Mrs Smith’s and her husband’s own descriptions that provide a degree of certainty to the present absence of AD.

At one moment, changes in Mrs Smith’s memory are related to the biophysiological changes in her brain. But this somaticization of Mrs Smith’s problems is only partial. Mrs Smith’s memory issues are made present through the assemblage of biomarkers *alongside* social processes (her history, her age, accounts of her behaviour and memory issues). She and her husband are made present as agentic subjects who have consciousness and who more importantly need to have knowledge – which Rose and Novas (2005) help us to understand is one of the cardinal characteristics of contemporary personhood.

In the memory clinic, processes of somaticization require connections to be made that align the differentiation of patients *as* persons who can be questioned, with objects that are being made to represent the presence of neuro-pathology. In this current case, this involves a complex notion of plasticity (reserves), previous pathology (stroke) and neurological deficit due to ageing (shrinkage and dark rims). The very objects (the brain scan, the cognitive test score) that are assembled to give dementia form also rely upon the invocation of subjects (the patients themselves, together with their families), whose participation as minded persons is paramount in the interpretation of the significance of behaviour, cognitive capacities and social as well as medical histories.

## Boundaries and resistances: Mobilizing patients as minded persons in the diagnosis of Alzheimer’s disease

The clinic, as Latimer (2013) has shown, is both a site of gathering (of persons, materials and heterogeneous forms of evidence) and a nexus of crossings between the world of developing biomedical sciences and the fleshy, social worlds of bodies, families and persons. Much like the collective production of uncertainty (Moreira et al., 2009) in the interface between biological sciences and the clinic, the clinic itself is a site in which the uncertainties of a dementia diagnosis provide a valuable resource for pragmatic action (Street, 2011). Although the brain’s pathology is brought into play in the clinic (as seen in the example above) to accomplish or exclude a dementia diagnosis, clinicians also engage in an assessment and evaluation of ‘minds’.

In this extract, Mrs Grayson and her son are attending the memory clinic for the first time. The exchange illustrates that the diagnostic process entails negotiations over entry into or out of a category, including mobilizing the patient herself as someone who is able to enter into negotiation. Following completion of a set of cognitive tests, Dr Summer discusses the results:

Doctor Summer: No. I – the aim of the exercise is saying, well are we within the normal ageing process or are we a bit beyond that? Also, what is important, yes we don’t remember as much as when we were 20 years younger. But the point is, how much impact is it having on your life? So obviously, yes, perhaps you are slightly under the cut-off point, the cut-off point on the score out of 100, we consider the cut-off point about 80 and you scored 76, you see.

Mrs Grayson: Cut-off point meaning what?

Doctor Summer: Well, when you were below that point, you suspect that you’re having a bit of a problem, a bit more significant problem. Above 80 you can consider it that well …

Mrs Grayson: Right.

Doctor Summer: You would consider how good you were and that sort of thing. So it’s all relative, you have to interpret things. I think that your brain scan, the scan is not diagnostic of anything, it could guide you a little bit towards something or the other. There is a little bit of a shrinkage of the brain. It says more, yes, more specific areas of the brain where the memory is, the hippocampus, we call it, and there is a bit of narrowing of the arteries, diffusely- so it means there is a bit less blood supply, oxygen supply to the brain. So that slows down – the shrinkage and the not 100% circulation could obviously account for the memory not being as sharp as it used to be.

Mrs Grayson: No different to the average people then?

Doctor Summer: Oh no, there are a lot of people a lot better than you. And lots of people are worse than you.

Mrs Grayson: Oh, no they’re not, they’re not.

Doctor Summer: But you scored 76, you can score up to 100, you see. So there will be old people scoring between those two figures. Yes, even people of your age can score better.

The object brought into play and around which the encounter plays out is the cognitive test score. The clinician works hard to persuade Mrs Grayson that her lapses in memory are not only due to the ‘normal’ ageing process, including marshalling her cognitive score as evidence of difficulties that breach the ‘normal’ neurodegenerative decline associated with age. Dr Summer also brings another object into play – the visual representation of the CT scan, referring to aspects of shrinkage located in the memory area of the brain, to strengthen her assertion.

Mrs Grayson explicitly rejects attempts to somaticize her experience. In countering this resistance, Dr Summer calls upon the tangible resources of the cognitive score and the image of the brain scan to help substantiate the pathological basis of the patient’s memory decline. In order to navigate the conflicts and contradictions present in the different accounts of memory decline being proposed in this encounter, Dr Summer is required to associate Mrs Grayson’s own narrative, that gives form to the functionality of her mind, with the pathological change shown in the brain scan, reinforced as evidence through the cognitive score. Dr Summer is thus attempting to smooth out the competing narratives of age and disease being brought into play by Mrs Grayson’s resistance, by appealing to Mrs Grayson as a reasonable subject, who in the face of the evidence can and will be persuaded.

In these ways, patients in the clinic are being shifted between representations of their brain pathology (cognitive test scores, percentages, brain scans, etc.) as somatic and the mobilization of them as ‘minded’ subjects whose agency and reason can be appealed to. We suggest that this process can be understood as how objects are attached to patients as experiencing subjects, through invoking them as minded persons who can think about their bodies (especially their brains) as objects that can be known. Through these moves, clinicians assemble evidence for the diagnosis of brains (the soma) as the part of patients that are or are not suffering from disease alongside processes that re-enact them as minded persons – a conscious subject, capable of reason and agency.

Mrs Grayson is a borderline case for the clinicians. In contrast, in this final extract the diagnosis is one of Alzheimer’s Disease. Mr Jones had an initial assessment and a follow up appointment to review the results of a CT scan of his brain. Although these tests showed some changes, the couple were told to wait a while and see how things develop over 3-6 months. In this interim period, Mr Jones has also had a repeat CT scan of the brain:

Doctor Glass: What this scan is showing is degenerative change over the memory areas of the brain specifically. Also a little bit of small vessel disease. Alright, small vessel disease is basically a small amount of thickening of the small vessels of the brain.

Mr Jones: That’s old age I suppose.

Doctor Glass: That can show with age yes. It doesn’t say it’s severe or anything, it just says it’s there. So you’ve got a little bit of hardening of the arteries to the brain, but more specifically you’ve quite clearly now got some degenerative change over the memory areas. Have you been worried about this being any condition in particular?

Mr Jones: No.

Doctor Glass: Because I think what we’re seeing really is that your memory problems are progressing over time. They’re not progressing rapidly, fortunately, but they are progressing. And there is some degeneration.

Mr Jones: Yes. Age.

Doctor Glass: Now I think this is probably a little bit more than age. I think this is probably now tipped over into being a bit of Alzheimer’s Disease. Okay. So have you thought about that possibility?

Mr Jones: I have thought about it yes. It hasn’t worried me at all.

Mrs Jones: Are you saying that it could develop into Alzheimer’s?

Doctor Glass: I think it probably has, Mrs Jones, is what I’m saying. I think there’s a line you know. So I think there comes a point, particularly when you begin to see the changes on the CT scan that we’ve got enough information to say that this is probably Alzheimer’s disease.

Mr Jones: Do you think though – this is me. I’m born lazy. And things that I should have remembered I’ve remembered. Things that come and they’re gone, don’t worry me …

Mrs Jones: I think his long-term memory is good.

Mr Jones attempts to persuade the doctor (and maybe himself) that his problems are age-related rather than pathological, but his move is weak. As in the previous example when the clinician meets resistance, Dr Glass asserts that the brain scan offers a visual representation of the pathological changes associated with Alzheimer’s disease. This is a strong move. In our previous example, Dr Summer described the necessity to interpret scans and situate them in the context of the patient’s overall abilities. In that case, AD was not made solid enough to be given as a diagnosis. In contrast, Dr Glass explains that once you start to see changes such as these on the CT scan you can confidently say that this is probable AD. Drawing on a regime of truth in which the underlying cause of Mr Jones’ problem can be revealed, the significance placed on the scan, at least for a moment, helps to make Alzheimer’s concrete as a bio-clinical entity.

During interviews, most clinicians, including Dr Glass, described the limitations of the CT scan as a tool that could never be diagnostic without a clinical presentation. In contrast, the mobilization of the scan as material evidence, in the face of Mr Jones’s resistance, performs the scan as a definitive epistemic object, which in turn does important symbolic work. It does not just help to make sense of the complexities, adding credence and legitimacy to the diagnosis of AD, it also helps shift, if only for a moment, Mr Jones’s explanations for his memory problems away from the ‘normal’ ageing process or from character traits, such as laziness, and towards neurodegenerative disease and a diagnosis of AD. Reasserting the scan as definitive reconnects the patient’s account – Mr Jones’s own experience of forgetting (even if it is only the unimportant things that he forgets) – to a bio-object, the locating of his troubles as pathological changes in his brain.

Mr Jones, at this moment and in this context, accepted that his memory cannot be a matter of his personality. This process does not just rely on Mr Jones being reasonable and accepting the evidence before his eyes, but re-enacts him as exactly that – someone capable of reason, and of being persuaded by the strong grounds that the doctor brings into play. Thus, in the clinic, it is this careful and cautious reading of the interaction itself, and the patient and families’ participation in it, that forms an integral source of evidence for the fabrication of dementia.

## Concluding thoughts: Braining-up dementia and its limits

We began this article by showing the challenges that dementia poses, its elusiveness, uncertainties and contradictions, and the effort required to maintain dementia as a stable biomedical entity that can give rise to imagined futures, to be shaped and managed by governments, institutions, families and individuals. We noted the ‘imprecision’ in binding dementia as a workable diagnostic category to epistemic objects – namely the biomarkers amyloid and tau and inflammatory markers. We have illustrated how scientists in our study attempt to navigate and even utilize dementia’s uncertainty, and the imprecision of associated categories like MCI, to smooth out the contradictions that threaten the epistemic authority of dementia science, especially the relation between brain, mind and personhood, through processes of somaticization. Somaticization, as a practice of giving dementia a material, bodily presence, represents attempts to traverse these necessarily contingent categories (Moreira et al., 2009).

In shifting our focus to the clinic, however, we have shown how the somaticizing of dementia that occurs in scientific research contexts is not and cannot be totalizing in the making of bio-clinical entities. Here our research echoes that of Boenink (2016), who asserts the ‘messy reality’ of dementia diagnosis, including stressing the need for the enhancement of both epistemic and translational responsibility. But we want to press a different finding. Like Boenink (2016) we want to show the messiness, but we also want to suggest that it is by examining the making of dementia across science and research and clinical diagnosis that we are able to illustrate the *limits of somaticization*.

We have shown that this somatization runs into problems in the clinic. The same clinician-scientists putting biomedical effort into firming up biomarkers such as amyloid and tau are also the clinicians or close colleagues and collaborators of the clinicians working in our two memory clinics. While dementia is being stabilized in research contexts through its somaticization, this approach seems to come unstuck in the clinic, as diagnostic processes do not merely invoke but seem to rely upon bringing the patient and their families into play as ‘minded persons’.

Our work parallels that of others who have shown the multiple meanings attached to dementia in memory clinics, and the ways in which these meanings can conflict, requiring diverse practices of mediation, even negotiation (Swallow, 2016). For example, Moreira’s (2010) work shows the continual shifts that occur between a regime of truth on the one hand, which seeks to assign presenting problems to biological changes in the brain, and a regime of care on the other, which instead attends to people’s specific situations and the ‘workable re-arrangements’ required for carrying on with life (Moreira, 2010: 132).

However, in this article we have shown how the subjects of dementia are and are not attached to biomarkers and other forms of objective evidence. Specifically, we have shown how and when various materials and sources of evidence, including CT scans of the brain, cognitive scores and medical histories, are associated with patients’ narratives of their thinking and memory, and the accounts of family members, through which a diagnosis of dementia can be firmed up, or not.

Critically, the social processes instituted in the clinic invoke the patient as a subject: a person capable of negotiation, of being reasonable, of being persuaded by evidence. In other words, in the clinic the person who may have dementia is also enacted as ‘minded’. Thus, in the clinic dementia is being fabricated through the association of ‘somatic’ evidence with evidence elicited in social processes. Even a person eventually diagnosed as ‘having’ AD, such as Mr Jones, is engaged in the clinic as a minded subject. This raises problems when early detection relies upon somatic rather than other forms of evidence. In the clinic, we have seen how patients are mobilized in ways that enact them as not just having agency, not just as someone who needs to be persuaded about the state of their body, but as someone who is reasonable enough to engage in negotiation and forms of persuasion that mobilize evidence of dementia. It is by associating these multiple sources of evidence and processes – including the artfulness with which clinicians proceed with caution, perhaps reading the interaction itself as a form of evidence – that clinical medicine stabilizes dementia as a disease, perhaps fleetingly a coherent bio-clinical entity, a ‘thing’ that can be treated, cared for or managed. As our interview extracts reflect, these practices can be identified not just for those who are deemed at risk of dementia or with the early stages of dementia, but also for those who have established dementia, as is the case for Mr Jones.

As discussed at the outset, governmental strategies pressing early detection and new thinking in the neurosciences align to locate dementia inside the brain as biomarkers and patho-physiological processes prior to its clinical presence. As the scientist we quoted put it ‘(T)hey don’t have dementia. We’re trying to prevent dementia.’ Somaticizing dementia is one particularly potent method through which early detection of dementia is being made concrete as a stable and coherent ‘thing’, providing the possibility of a fixed target at which science, medicine and governments can take aim. But somaticization of dementia is not as totalizing at it seems. Rather, as we have shown, in the clinic patients and their bodies need to ‘bear witness’ in some way to make dementia as a bio-clinical entity stable.

Critically, in clinical contexts, epistemic objects such as biomarkers are treated as ‘provisional’ (Boenink, 2017). Indeed, we suggest, dementia as a bio-clinical entity needs the clinic – as a site in which mind, personhood and body can be held apart one moment, and through the assembling and associating of different forms of evidence, such as the brain scan that visualizes ‘pathology in the corporeal interior’, collapsed into soma the next. Here the personhood of the patient themselves is brought into play, with the doctor ‘bearing witness’ (Charron, 2006) to their capacity to engage in interaction as, perhaps, itself a form of evidence. These clinical practices reflect moments in which a medical model of dementia as progressive may be disrupted, allowing for a less linear and more complex picture to emerge. For example, the case of Mrs Smith prompts a discussion of brain reserves and the brain’s capacity for compensation.

In the light of government funding being directed at the prevention of dementia, we have in this paper pointed to the difficulties in relying on evidence in the form of bio-markers alone. Such a position over-somaticizes the condition of dementia as if it exists as a continuum of brain degeneration. Early or mild forms of dementia, by their very definition, involve forms of self-consciousness that exhibit reliance on the evidence of minds. Our study of memory clinics clearly shows how the accounts of patients and families, and their mobilization as participants in situation-specific processes of negotiation and persuasion, are integral to the intermittent and precarious stabilization of dementia as a bio-clinical entity. In so doing, we are pointing to the irony of this finding: that the stability of dementia diagnosis in the clinic may require patients to perform themselves as minded persons, capable of negotiation and persuasion through participation in reasoned argument, even as at the same time, as in the case of Mr Jones, they are suspected of having ‘a bit of Alzheimer’s Disease’.
